# Zika Virus Infection during Pregnancy and Congenital Abnormalities

**DOI:** 10.3389/fmicb.2017.00581

**Published:** 2017-04-04

**Authors:** Irfan A. Rather, Jameel B. Lone, Vivek K. Bajpai, Yong-Ha Park

**Affiliations:** ^1^Department of Applied Microbiology and Biotechnology, School of Biotechnology, Yeungnam UniversityGyeongsan, South Korea; ^2^Department of Biotechnology, Daegu UniversityGyungsan, South Korea

**Keywords:** ZIKV, microcephaly, infection diseases, outbreak, mosquito

## Abstract

The presence of the Zika virus (ZIKV) infection has gone ahead to be a threat to people based on its adverse impacts. More specifically, the pregnant women have been discouraged from traveling to the areas affected by the ZIKV because of the likelihood of the virus causing congenital abnormalities especially the microcephaly. The pregnant women probably attracted the virus during their first trimester while visiting ZIKV affected territories. Although the ZIKV infected cases have reduced in some parts of countries, the global risk assessment has not been changed. The virus continues to spread geographically to areas where competent vectors are present. At present, there is still no treatment of ZIKV related illness, especially microcephaly.

## Overview

The Zika virus (ZIKV) was discovered during a study conducted in 1947 aimed at establishing the causative agents of yellow fever, dengue, and other West Niles viruses (Dick et al., 1952; Korhonen et al., 2016). The virus was first identified in the blood of a sentinel rhesus monkey in Zika Forest in Entebbe, Uganda (Shors, 2016). The virus was later discovered in a field worker and then spread sporadically to other humans in Africa and Asia and continued to remain insignificant for over six decades from when it was first isolated. It was until its outbreak in Brazil that its spread accelerated rapidly throughout the Americas and other parts of the world raising global health concerns. The increase in the number of women infected with the ZIKV giving birth to children with suspected congenital defects raised fresh concerns by health organizations to research on possible links between the ZIKV infections and brain related defects to the infected mothers during prenatal and postnatal stages of pregnancy. Extensive research was vital to be able to obtain factual proofs rather than mere speculations regarding the seriousness of the infection and on the fetuses and subsequent health status of the newborn babies after the infection. Currently, there are several countries with active ZIKV.

### Transmission Areas

These include Aruba, Barbados, Bolivia, Brazil, Colombia, Costa Rica, Cuba, Ecuador, Mexico, Guatemala, Haiti, Panama, Trinidad and Tobago, and American Samoa among others (Centers for Disease Control [CDC], 2017a). Therefore, pregnant women should not travel to areas with active ZIKV transmission. Several studies in the ZIKV affected countries have established adverse effects of the virus on the neonates leading to the bearing of children with various malfunctions associated with the presence of the ZIKV among the pregnant mothers (Centers for Disease Control [CDC], 2016b). The commonly known birth defect covered in this review linked to the presence of ZIKV giving birth to children suffering from microcephaly. Microcephaly is a brain abnormality characterized by giving birth to children whose heads are abnormally smaller than normal babies irrespective of sex and age (Magauran et al., 2016; Marcondes, 2016). This review is purposely meant to unravel the association between the ZIKV infection during pregnancy, and congenital abnormalities exhibited in children with the virus (McNeil, 2016; Posen et al., 2016). A clear and a deeper insight of the existing relationship can only be achieved through a critical analysis of varied studies about the ZIKV and congenital abnormalities. Therefore, an effort was made to obtain factual information regarding the links between the ZIKV infection and birth-related defects and discard speculative cases of ZIKV as a global menace. Besides, the review assesses the known modes of transmission of the virus from the infected pregnant mothers to the fetuses and how they lead to related brain defects. However, some of the reports have also been cited to discredit ZIKV as the sole cause of all brain damages affecting children before and after birth (Clammer, 2016). Some of these causes of brain damages also include genetic and physical interaction with the toxic chemicals (Jaishankar et al., 2014). The main focus of this review has been put on microcephaly as one of the common congenital birth defects believed to be caused by ZIKV. In most babies, the causes of microcephaly are unknown. State birth defects tracking systems have estimated that microcephaly ranges from two babies per 10,000 live births to about 12 babies per 10,000 live birth in the US (NBDPN, 2013).

The ZIKV is an arthropod-born virus of the Flaviviridae family and is transmitted by the bite of several *Aedes* mosquitoes (Anderson et al., 2016; Focosi et al., 2016; Musso and Gubler, 2016; Petersen et al., 2016). The virus can also be transmitted through the placenta transplant (Besnard et al., 2016), blood transfusion (Venturi et al., 2016), and sexual activities (Hills et al., 2016). The spread of ZIKV remained confined within some specific parts of Sub-Saharan Africa, South East Asia and the America during the 1940s. The confinement of the virus in few countries can be interpreted to mean the high level of reluctance by the World Health Organization (WHO), making countries like the Americas to underestimate its impacts due to insignificant effects on its population before 2015 when it got into Brazil and the Pacific areas. However, the situation changed due to the increasing cases of infants with microcephaly who were born from mothers infected by ZIKV raising the need to carry out research to establish the link between the virus and other birth related defects (Magauran et al., 2016).

## The Associations Based on the Trends of ZIKV Spread

Pan American Health Organization and World Health Organization establish that the first case of ZIKV infection in the human was discovered in 1954 in Nigeria (MacNamara, 1954). Up to this time, the virus was perceived as a preserved issue of African and some parts of Asia. However, this notion changed in 2007 when the pandemic befell Micronesia and Island in the State of Yap with over 5000 infections in a population of 6700 (Duffy et al., 2009). Another outbreak of the infectious virus was reported in French Polynesia in 2013 and 2014 (Hancock et al., 2014). Other related cases also occurred in 2014 in Pacific Islands like in Cook Islands, Samoa, and American Samoa (Hancock et al., 2014; Tappe et al., 2015; Magauran et al., 2016). The increasing trend in the spreading virus indicated how this dangerous ZIKV infection was able to cause inevitable trouble that the world was likely headed to if the problem was not controlled in time. Consequently, the concomitant increase in the infection of the virus and the cases of microcephaly raised more questions about the association between ZIKV infection and congenital abnormalities among the infected pregnant women (Musso, 2015; Barcellos et al., 2016). The existence of this virus in the Americas was first identified in March of 2015, when an outbreak of an exanthematous illness occurred in Bahia, Brazil (Campos et al., 2015; Zanluca et al., 2015). By October, 2015, ZIKV had spread to at least 14 Brazilian states, and in December 2015, 1.3 million suspected cases were reported by the Brazil Ministry of Health (Hennessey et al., 2016). By September 2015, a tremendous increase in the number of cases of children born with microcephaly was reported, in the areas in which ZIKV was first reported (Schuler-Faccini et al., 2016). The unabated steady increase in the related cases of microcephaly indicates that minimum attempts have been made to curb more effects of the deadly ZIKV. The initial discoveries of the virus were identified from two pregnant Brazilian women whose amniotic fluid tested positive for the ZIKV and were later established in the fetuses which further confirmed the possible relationship between the ZIKV and the causes of microcephaly. More research was imperative to have more samples to choose from and strengthen the previously established links. An extensive research commissioned by the Brazil Ministry of Health served to the purpose of building on the data that had already been carried by other studies of the infection of the virus and congenital defects among the unborn. The research examined the children suffering from microcephaly and how their mothers might have been affected by living or visiting countries with ZIKV during pregnancy. There was a common result obtained from the 35 infant cohorts used during the study (Schuler-Faccini et al., 2016). They all had the lumbar puncture, a situation which made researchers to suspect a link between this defect and the ZIKV. However, clear results were not obtained from the Brazilian Laboratory to conclusively argue that the ZIKV causes microcephaly in children and such brain damages as discovered on the lumber, making it hard to base on this outcome as a clear proof of linkage. The potential relationship that is aimed at in this review is largely based on supported clinical results to ascertain the associations.

There are also statistical data that have been presented to confirm the link between the ZIKV and child birth defects. For example, the epidemiological report for monitoring cases of microcephaly in Brazil established that about 75% cases of microcephaly were reported on February 6^th^, 2016 which were still under investigation. About 62% of these cases were reported in 2015 and over 37% in 2016. Of all investigations conducted, 15% of the children diagnosed indicated the presence of microcephaly, which was characterized by changes in the central nervous system suggesting an instance of congenital infection in the children. The report also confirmed that out of the total number of the investigated cases expressing congenital infection, a considerable percentage was identified to have ZIKV through an extensive clinical laboratory test conducted (Clammer, 2016; Naccache et al., 2016). These cases can be used to justify a link between the ZIKV infection and congenital infection, though the actual numbers of children born with microcephaly associated with the ZIKV have not been established.

Effectiveness can be achieved if the association is further broken down into modes of transmitting ZIKV from the environment to the unborn and how the infection can impact on the unborn regarding the brain development. The symptoms of the infection have also been briefly examined to help identify the children with ZIKV related brain infections.

## Transmission of the Virus

The ZIKV is largely transmitted through a bite by an infected mosquito of the *Aedes* genus majorly found in the tropical areas (Anderson et al., 2016; Focosi et al., 2016; Musso and Gubler, 2016; Petersen et al., 2016). These mosquitoes are similar to those believed to transmit dengue and yellow fever (Sikka et al., 2016). ZIKV transmission is also believed to occur through sexual activities (Hills et al., 2016). The means have been considered a matter of concern due to the perceived adverse impact of the ZIKV on pregnancy and the possible effects on the fetuses (Naccache et al., 2016; Nicastri et al., 2016). In areas with high transmission rates, infected people can transmit the virus to their partners. It is, therefore, recommended that men and women undergo counseling to know the right time to conceive to avert problems during pregnancy and the deadly outcomes of the ZIKV on the child during pregnancy. Besides, it is imperative that women who are interested in becoming pregnant, but fear due to the risks of ZIKV should have protected sex or use contraceptives (Nicastri et al., 2016). Safe sex and abstaining should be practiced by mothers during pregnancy for women in areas with active transmission of the virus. In addition, it is recommended that safe sex be practiced or abstinence adopted for men and women who are returning from ZIKV active transmission areas to safe regions to prevent the transmission through sexual intercourse. Transmission through this mode occurs before the symptoms of the illness are realized or during the development of the symptoms. However, these discoveries are limited because they do not establish clear risk-factor duration.

Various evidences have been shown to indicate the possibility of transmitting ZIKV from the mother to the fetuses during pregnancy (Centers for Disease Control [CDC], 2017c). ZIKV RNA was identified in the brain tissue of children with microcephaly who were later reported to have died after their delivery (Branswell, 2016). Studies have shown that the presence of this virus can be detected through ultrasonography by examining the amniotic fluid of the mothers.

There are also suspected possibilities of the virus being transmitted through blood transfusion (Venturi et al., 2016). The assumption here is that other flaviviruses are also transmitted through the same route (Hancock et al., 2014; Nicastri et al., 2016). There is limited research that has been done to affirm this truth. The test carried during the French Polynesia established that about 3% of the blood tested had tested positive for the ZIKV.

Monkey bite can also lead to the transmission of the ZIKV. Such a case was identified in Indonesia. It is also suspected that the ZIKV can be transmitted through breastfeeding from the women who have the symptom of the ZIKV during delivery (Bradford, 2016). Such cases are possible if the ZIKV is highly concentrated in the body of the carrier who might, in turn, infect the child. At present, no cases of ZIKV infection associated with breastfeeding have been reported (Centers for Disease Control [CDC], 2017b).

## Symptoms in Pregnant Women and Potential Evidence

**FIGURE 1:**
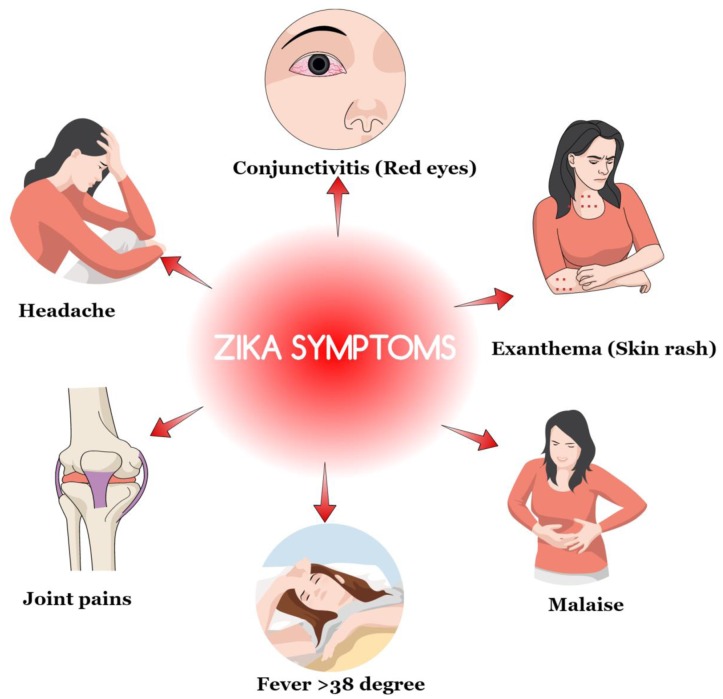
**Common noticed symptoms of Zika virus (ZIKV) infection**.

According to the WHO, the time between exposure and the symptoms of the ZIKV have not been clearly identified. However, the research assumes that the incubation period is likely to be a few days similar to other related arbovirus infections like dengue. Moreover, over 80% of total women who were infected by ZIKV showed the minimal clinical manifestation of symptoms (Bradford, 2016). The commonly noticed symptoms include fever (>38°C), skin rashes, joint pain, malaise, conjunctivitis, headache and fatigue, myalgia, abdominal pain, and vomiting among others (**Figure 1**). Some women had reported rashes that were seen on the faces, palms and soles. However, several studies have also noted that not all these symptoms present in women were infected by the ZIKV. Some of the cases tested showed that some women who were infected had no physical signs (Bradford, 2016; Centers for Disease Control [CDC], 2016c). It can, therefore, be justified through these studies that some of the non-clinical signs are obtained through the principle of generalization raising possible doubts about the universal physical identification of the infected personalities.

## Microcephaly

**FIGURE 2:**
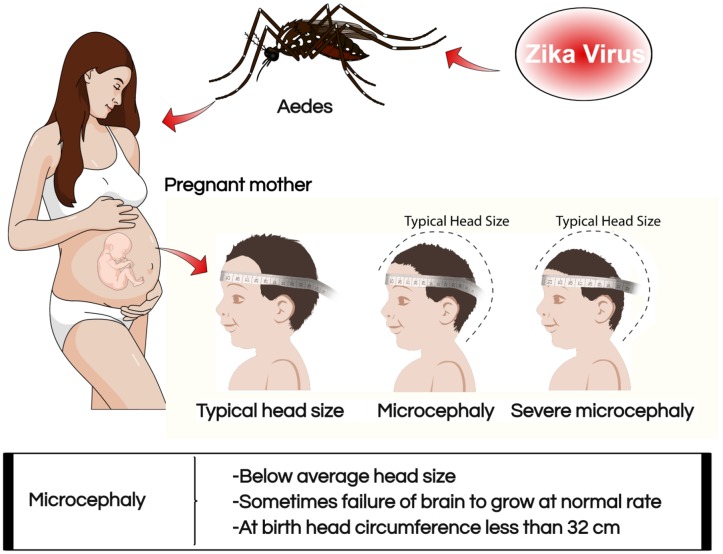
**Association of ZIKV with microcephaly in pregnant mothers**.

Zika virus has been linked to some of the birth defects experienced by mothers who were infected with the virus during their pregnancy (Centers for Disease Control [CDC], 2017b). The most commonly noted birth outcomes associated with the infection include microcephaly and related brain problems in infants (Campos et al., 2015; Barreto de Araújo et al., 2016). The Center for Disease Control and Prevention defines microcephaly as a birth defect where a child is born with small sized head caused by ZIKV (**Figure 2**). Biologically, the growth and development of the brain happen concurrently with the head. The condition occurs when there is an inadequate development of the brain during the prenatal stage in the head and causing the brain to stop growing after childbirth. The condition can occur in isolation or occur together with other congenital defects prevalent among the fetuses and babies who have been infected by the virus during pregnancy (Jones et al., 2016). Severe microcephaly is characterized by seizure, delay in the development of speech, siting, standing and walking problems (Centers for Disease Control [CDC], 2016a). Children with the condition also experience difficulties in intellectual development which result into decreased learning abilities and influence the normal functioning daily life (Mlaka et al., 2016; Schuler-Faccini et al., 2016). They also experience feeding problems, hearing loss, and vision impairment. A severe condition of this defect is deadly and can only be managed through frequent check-ups and health care monitoring services since they are not treatable. There are two forms of microcephaly like, congenital and acquired (Nicastri et al., 2016). Congenital microcephaly is genetic and is passed from one member of the family whose brains had been infected by the virus causing the defect to the siblings. Acquired microcephaly refers to the case where the brain of the fetus comes into contact with a harmful situation altering the normal growth and development. Fetuses risk acquiring microcephaly in the womb when the pregnant mothers impaired infectious condition specifically with viral infections caused by rubella, chickenpox, and ZIKV. However, other toxic malnutrion and infectious conditions such as hemorrhage state, stroke, and brain injury may also lead to fetus acquired microcephalySevere microcephaly is a considered a feature of congenital ZIKV syndrome (Barreto de Araújo et al., 2016). An infant with congenital ZIKV infection was born without showing any signs of microcephaly; however, later experienced slow growth of the head (Van der Linden et al., 2016). The condition is described as a postnatal microcephaly, which is equally dangerous in the life of the child. These causative agents of microcephaly are important in broadening the understanding of the causes of brain problem. It is, therefore, possible that non-clinical tests can result in a mistaken case of suspected ZIKV on the neonates based on mere observation on physical symptoms.

Furthermore, results from the studies published in the New England Journal Medicine discovered more relationships between the ZIKV infection and the causes of microcephaly and brain anomalies in newborn babies (Michael et al., 2016). The assessment criteria of potential teratogens were used in evaluating the existing data on pregnant mothers. The research coorelated the findings on accumulating evidence to support the existing links of the virus microcephaly during the prenatal development. No research practices were performed making the outcome dependent on the availability of past studies which were accumulated (Centers for Disease Control [CDC], 2016a; Costello et al., 2016). Consistency in the observed defects, was associated with brain anomalies that caused by the presence of ZIKV obtained from the brain tissues of the infants or the fetuses, further supporting the link between ZIKV infection and microcephaly (Kaslow et al., 2014; Rasmussen et al., 2016). This research failed to mention a direct link between the infections rather that accumulating relevant data on the causes of the microcephaly among infants. However, the research provides precautionary measures aimed at preventing the possible adverse outcomes of the confirmed cases of ZIKV infection. Nevertheless, no clear risk factors are identified of the virus on the fetuses during pregnancy and the various stages of infection to reduce the burden of ZIKV effects.

Wang and Ling (2016) established causal links between the ZIKV infection and microcephaly. The article by the duo stressed on the previous research outcomes which showed the unusual increase in the number of cases of microcephaly as identified in Pernambuco and their association with ZIKV (Olusanya, 2011; Michael et al., 2016). However, the article adds nothing new to the existing data about the expected cases discovered in Brazil. The article suggests that the infection by the ZIKV can be detected through a standard fetal ultrasound scans on the brain of the fetuses. Fetal abnormalities can be discovered through an ultrasonography among pregnant women who are not showing signs of the infection of the ZIKV. The process can identify factors that deter the normal growth and development of the brain and the subsequent microcephaly which is associated with the Zika virus. The virus is also deadly because it can affect the Central Nervous System, thereby leading to the death of the infant (Nicastri et al., 2016). The article stresses on the effectiveness of using scan in identifying congenital ZIKV syndrome symptoms. A case about two women who were diagnosed of ZIVK can also be used to establish a link between the cause of microcephaly and the ZIKV (Schwartz, 2016a). When the women gave birth, they miscarried, and autopsies were performed on the two fetuses in both cases. Various parts of the head, other organs of the body, such as lungs, heart, skin, spleen, thymus, kidney and the brain tissues were tested for the presence of ZIKV (McNeil, 2016). It was realized that the fetus whose mother had tested positive for the ZIKV, had traces only in the brain tissue. However, the report is discredited for lack of direct proof of an existing link between the ZIKV and the cause of microcephaly (Shapshak et al., 2015). However, the article is vital in strengthening the link between ZIKV infections and how it leads to congenital birth defects and possible damage to the brain (Zanluca et al., 2015). The reports about this case also indicated that placenta of infected mothers and brain tissues can be used to further understand the link between the means of transmission during pregnancy to the unborn.

The other studies reported also examined the possible link between ZIKV infection and congenital birth defect in the case of microcephaly, which is a common type of brain abnormality believed to be caused by the ZIKV (Microcephaly Epidemic Research Group, 2016; Wang and Ling, 2016). The report noted that the effects of microcephaly depend on its exposure, which can be mild or serious depending on its long-term exposure on the brain, which can be mild or serious in the development of the brain. When the brain is mildly damaged, the child can experience slow development in the brain functions while when it is serious, the child’s intellectual, motor can be adversely affected (Simões et al., 2016; Southwell et al., 2016). The study also ascertains other than congenital infections, chromosomal abnormalities like exposure to illicit drugs, craniosynostosis, which result from the premature fusion of bones of the skull and other metabolic disorders (Campos et al., 2015). The research fails to single out ZIKV as the main cause of microcephaly because there are several related brain damages which have varied causative agents making it hard to get a clear distinction of ZIKV as the sole cause of microcephaly as a congenital infection of the fetuses. Therefore, for areas, which have been considered to have active transmission of Zika virus, attempts should be made to deal with the spread of mosquitoes to eliminate these possibilities of infections. These are considered both personal and national protective measures that can reduce cases of ZIKV infections that results from ZIKV spreading *Aedes* mosquitoes.

## Conclusion

In this review, an effort was made to unfold a close link between ZIKV infection during pregnancy and congenital abnormalities. The first confirmed evidence of this association is based on the Pan American Health Organization and World Health Organization reports which updated on the status of the ZIKV infection based on the discoveries made in Brazil and French Polynesia in 2015 and 2014, respectively. However, these studies were never based on direct factual proofs, but relied on fetal malformations which were associated with the infection of ZIKV (Hancock et al., 2014; Campos et al., 2015). The related abnormalities which were linked to this infectious virus were the occurrence of several cases of microcephaly and other nervous system defects on the fetus among pregnant mothers who were diagnosed with ZIKV. Moreover, pathologists have also helped to strengthen the link by examining brain tissues of children who contracted the infection from their mothers during pregnancy through autopsy (Schwartz, 2016b). Moreover, the environment also plays a role in the transmission of the ZIKV. For instance, traveling partners from the areas where the ZIKV infection could transmit through sexual intercourse (Harrower et al., 2016; Plourde and Bloch, 2016). It is, therefore, important to refrain from unprotected intercourse when such cases are suspected within a particular area. However, all the research covered in this review fails to present how one can be completely protected from the risks of the ZIKV infection. Other than the precautionary measures recommended, there are no treatment mechanisms suggested or medical preventive mechanisms like vaccination presented for future safety. Moreover, there is little much direct evidence presented to directly link ZIKV with congenital abnormalities other than microcephaly, which is a single case of brain and head defect. Therefore, there is a need for more research for conclusive outcomes that can be utilized to curb the spread of ZIKV and reduce global health fear about the spread of this infectious virus.

## Author Contributions

IR wrote the initial draft, JL and VB designed the manuscript, Y-HP did critical analysis and approved the paper.

## Conflict of Interest Statement

The authors declare that the research was conducted in the absence of any commercial or financial relationships that could be construed as a potential conflict of interest.
